# Immunoglobulin M (IgM) multiple myeloma versus Waldenström macroglobulinemia: diagnostic challenges and therapeutic options: two case reports

**DOI:** 10.1186/s13256-020-02380-2

**Published:** 2020-06-22

**Authors:** Simona Elba, Alessia Castellino, Roberto Soriasio, Claudia Castellino, Margherita Bonferroni, Daniele Mattei, Giuliana Strola, Daniela Drandi, Nicola Mordini, Miriam Foglietta, Davide Rapezzi, Ivana Celeghini, Mariella Grasso, Fabrizio Giordano, Giulio Fraternali Orcioni, Massimo Massaia

**Affiliations:** 1 Azienza Ospedaliera Santa Croce e Carle, Via Michele Coppino, 26, 12100 Cuneo, CN Italy; 2 Department of Molecular Biotechnologies and Health Sciences, University of Turin, Turin, Italy

**Keywords:** IgM monoclonal gammopathy of unknown significance (MGUS), IgM multiple myeloma, Waldenström macroglobulinemia

## Abstract

**Background:**

Immunoglobulin M multiple myeloma and Waldenström macroglobulinemia are two different hematological diseases with the common finding of an immunoglobulin M monoclonal gammopathy of unknown significance. However, clinical characteristics of the two entities can overlap.

**Case presentation:**

In this report, we describe two cases of immunoglobulin M neoplasm with the same histological bone marrow presentation but with different clinical behavior, cytogenetics, and biological assessment. On the basis of comprehensive diagnostic workup, these patients were considered to have different diseases and treated accordingly with different approaches. Patient 1 (Caucasian man) presented with increased serum protein and immunoglobulin M (7665 mg/L) with an M-spike electrophoresis of 4600 mg/L. His bone marrow biopsy revealed a small-cell immunoglobulin M multiple myeloma. The result of testing for the MYD88 L265P mutation was negative, while fluorescence *in situ* hybridization analysis showed translocation t(11,14). A diagnosis of immunoglobulin M-κ multiple myeloma was made. Patient 1 was a candidate for bortezomib plus thalidomide and dexamethasone, followed by autologous stem cell transplant consolidation. Patient 2 (Caucasian man) showed an M-spike by protein electrophoresis (300 mg/L, 4.9%), with serum immunoglobulin M level of 327 mg/L. His bone marrow biopsy revealed immunoglobulin M-κ multiple myeloma. Computed tomography showed many enlarged lymph nodes and splenomegaly. Patient 2’s clinical features were suggestive of Waldenström macroglobulinemia, in contrast to the bone marrow biopsy results. The result of testing for the MYD88 L265P mutation was positive. Patient 2 was diagnosed with Waldenström macroglobulinemia and received rituximab, cyclophosphamide, and dexamethasone.

**Conclusions:**

A correct differential diagnosis between immunoglobulin M multiple myeloma and Waldenström macroglobulinemia is a critical point in the setting of a new immunoglobulin M monoclonal gammopathy onset. These patients should undergo a complete diagnostic workup with pathological, radiological, and serological examinations to establish the diagnosis and plan the most appropriate treatment in order to improve the prognosis.

## Background

Immunoglobulin M (IgM) multiple myeloma (MM) and Waldenström macroglobulinemia (WM) are two different hematological diseases with the common finding of an IgM monoclonal gammopathy of unknown significance (MGUS) [1]. IgM MM is a rare hematological disease representing less than 0.5% of all myeloma cases [1]. Differential diagnosis with other IgM-related entities, such as IgM MGUS and WM, still represents a challenge for clinicians.

IgM MM is characterized by the neoplastic proliferation of plasma cells preferentially in the bone marrow, producing a monoclonal immunoglobulin in the blood and/or urine [2]. Also, for IgM MM, likely other myelomas, CRAB (hypercalcemia*,* renal failure*,* anemia*,* and bone lesions) criteria, and myeloma-defining events (bone marrow plasma cells > 60%, kappa/lambda ratio ≥ 100, > 1 magnetic resonance imaging focal lesion) lead treatment decisions [1, 3].

WM is an IgM-secreting lymphoplasmacytic lymphoma that is more likely characterized by enlarged adenopathy, hepatomegaly and/or splenomegaly, anemia, IgM component–related symptoms such as hyperviscosity and peripheral neuropathy, and sometimes constitutional symptoms [3, 4].

However, sometimes clinical characteristics of the two entities can overlap, and a differential diagnosis can be difficult. A bone marrow biopsy is mandatory for a correct diagnosis, together with a complete histopathological, immunohistochemical, cytogenetic, and molecular analysis.

IgM MM is usually characterized by bone marrow plasma cell infiltrates that usually express surface IgM, CD38^+^, CD138^+^, MUM1/IRF4^+^, CD20^−^, CD19^−^, cyclin D1 positivity, and lack of CD56 and CD117. WM is characterized by IgM^+^CD5^−^, CD10^−^, CD11c^−^, CD19^+^, CD20^+^, CD22^+^, CD23^−^, CD25^+^, CD27^+^, CD10^−^, and CD138^−^ [3].

The presence of an MYD88 mutation is usually absent in IgM MM; in contrast, it is pathognomonic in WM [5]. The presence of translocation t(11; 14) detected by fluorescence *in situ* hybridization (FISH) is shown in IgM MM but is absent in WM [6]. Because of the overlapping of these clinical entities, the correct diagnosis is possible only on the basis of a complete evaluation with clinical, biological, and radiological tests.

In this report, we describe two cases of IgM neoplasms with the same histological bone marrow presentation but different clinical behavior and different cytogenetic and biological assessment results. On the basis of a comprehensive diagnostic workup, these patients were treated with different approaches.

We think that reporting these two cases is important to underline the need for a comprehensive, multidisciplinary diagnostic and therapeutic approach to these rare hematologic neoplasms. Because IgM monoclonal gammopathy is a pathognomonic sign of many different hematological diseases, all of these patients should undergo a complete diagnostic workup with pathological, biological, radiological, and serological examinations. In the two cases we report, the situation was even more complex because these patients with different diseases had the same bone marrow biopsy presentation. Moreover, higher importance is given to a precise diagnostic assessment because the availability of target biological drugs (such as ibrutinib or venetoclax) could represent a powerful therapeutic strategy if given to selected patients.

## Case presentations

### Patient 1

**Fig. 1:**
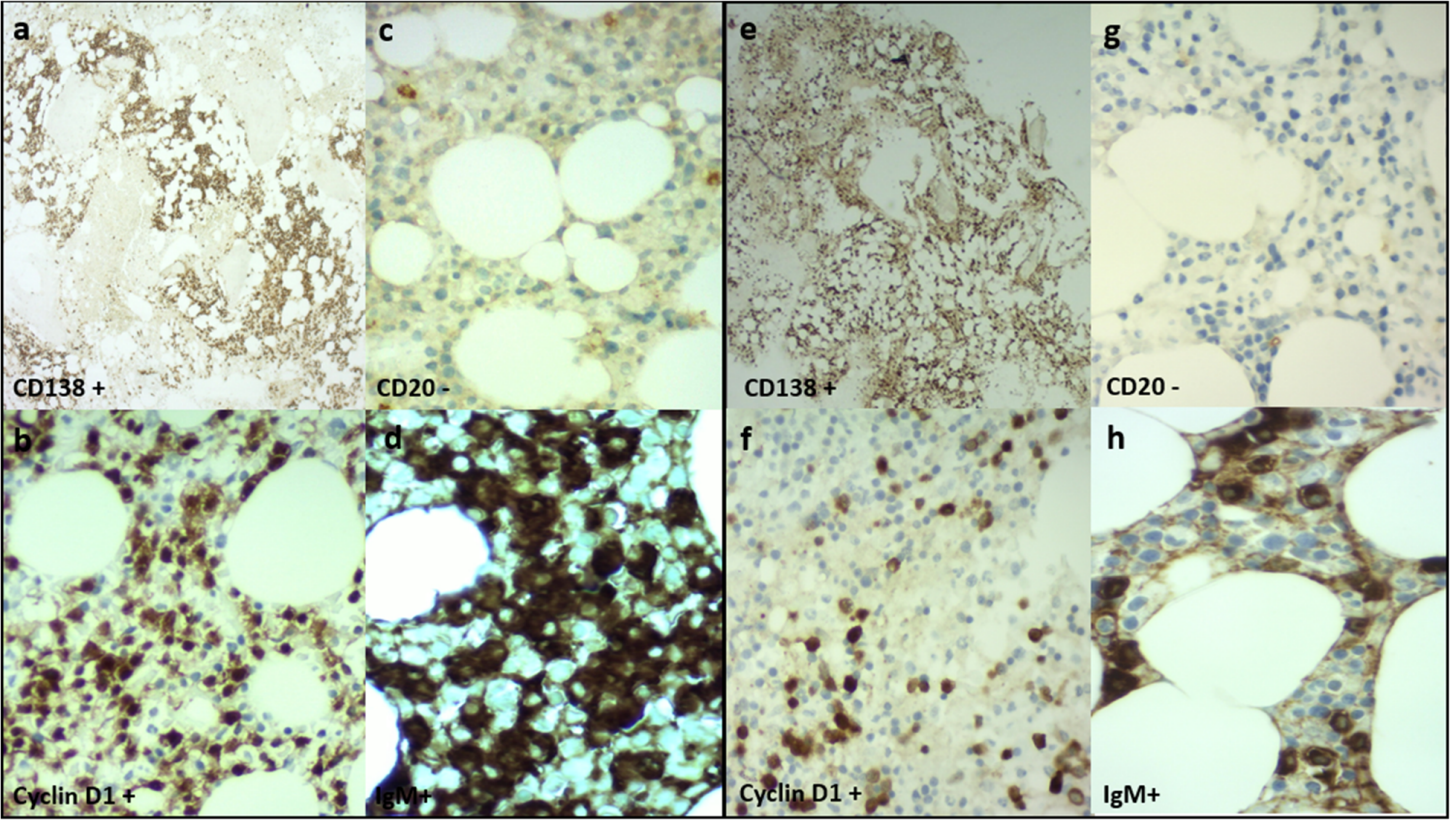
Bone marrow biopsy slides of representative immunohistochemistry. **a**–**d** Bone marrow slides of patient 1. **e**–**h** Bone marrow slides of patient 2

**Fig. 2:**
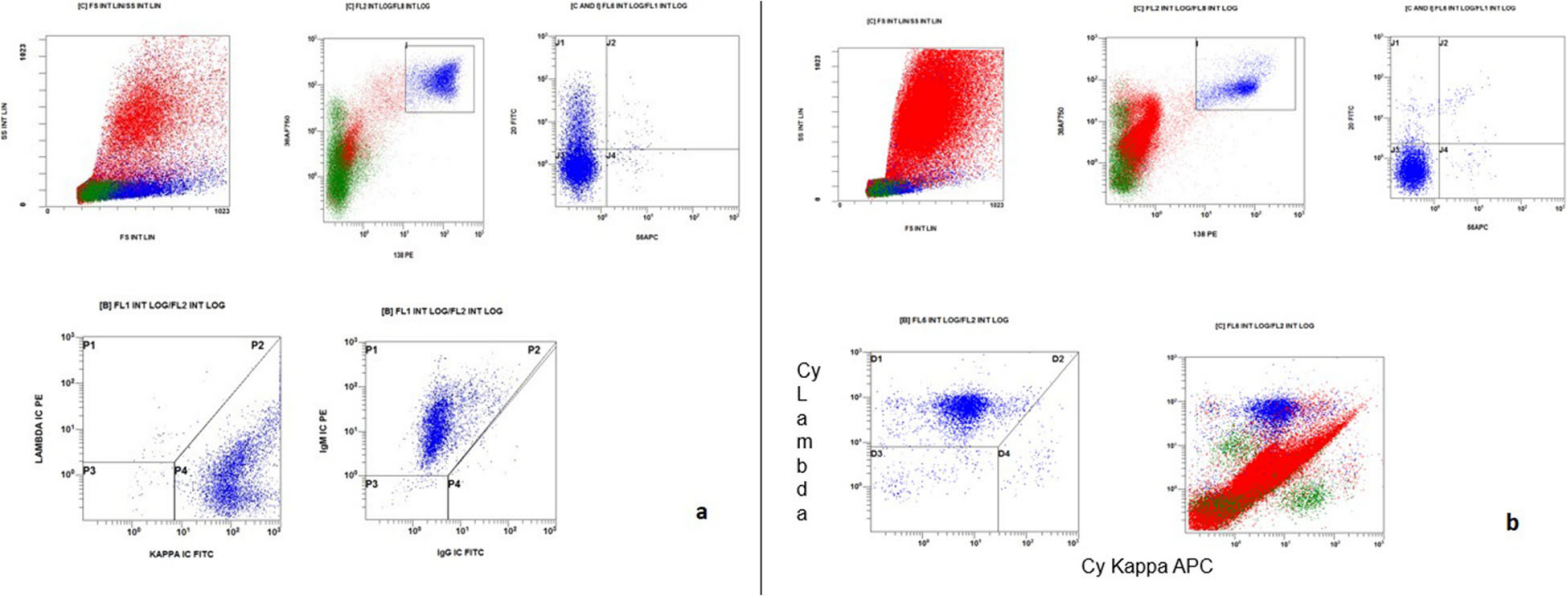
Flow cytometry showing representative flow in bone marrow analysis. **a** Patient 1. **b** Patient 2. The images were obtained with Navios, Backman Coulter flow cytometry

**Fig. 3:**
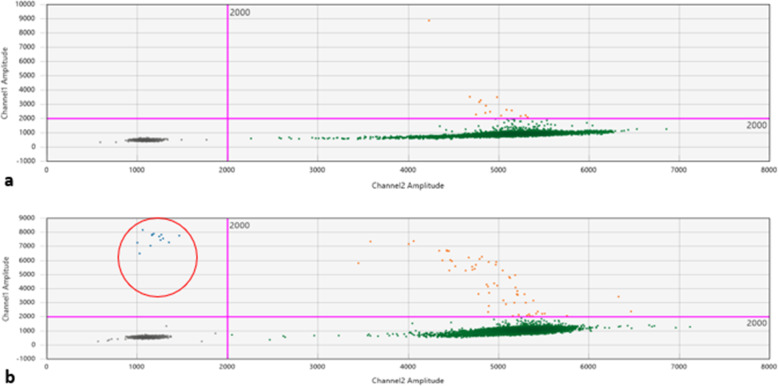
Digital polymerase chain reaction plot for MYD88 L625P mutation on bone marrow samples. **a** Patient 1, MYD88 result, wild type. **b** Patient 2, MYD88 L625P result, mutated

Patient 1 was a 70-year-old Caucasian man who was an only child. He was married with two daughters and was a retired employee. He had no positive history of allergy, was a smoker, and did not drink alcohol. He had undergone surgery for renal lithiasis at the age of 20 years and transurethral resection of the prostate 2 years before his presentation at our clinics. Otherwise, he had a silent medical history and did not consume any chronic medication before his hematological disease diagnosis. In August 2018, he experienced clinical onset of fatigue, headache, and arthralgia. His clinical examination revealed that he had no adenopathy or splenomegaly, no fever, no weight loss, no night sweats, and no new-onset bone pain. Upon admission, his blood pressure was 135/80 mmHg, pulse was 70 beats/minute with sinus rhythm, and body temperature was 36 °C. His neurological assessment did not show any neurological impairment. The initial laboratory test results showed remarkably increased levels of total serum protein (10,700 mg/L) and IgM (7665 mg/L), with protein electrophoresis showing an M-spike of 4600 mg/L (43.3%). His serum creatinine and calcium levels were normal, while a moderate macrocytic anemia was observed (hemoglobin [Hb] serum level 9.5 g/dl; while platelet and leukocyte counts were normal). His liver function was normal, but his β_2_-microglobulin was increased (3.15 mg/dl). A bone marrow biopsy with flow cytometry, immunohistochemistry, FISH, and MYD88 mutation analysis was performed. The bone marrow biopsy showed a small-cell IgM MM with an increased number of CD138^+^, CD79a^+^, free monoclonal κ-light chain restricted, IgM^+^, cyclin D1^+^, CD31^+^, CD45^−/+^, CD20^−^, EMA^−^, CD56^−^ plasma cells (65–70%) (Figs. 1a–d, 2a). Testing for somatic MYD88 L265P mutation showed a negative result (Fig. 3a). FISH analysis showed translocation t(11,14). His low-dose computed tomographic scan excluded lytic bone lesions and did not reveal evidence of any adenopathy or organomegaly.

Because the neoplastic clone was CD20^−^, CD56^−^, CD138^+^, and cyclin D1^+^ with the absence of a somatic MYD88 L265P mutation and with a positivity for t(11,14), and in consideration of the patient’s clinical presentation (absence of adenopathy, organomegaly, and B-constitutional symptoms), a diagnosis of WM was unlikely, and IgM-κ MM was finally defined. On the basis of the patient’s good performance status and favorable comorbidity scale, he was a candidate for induction therapy followed by autologous stem cell transplant (ASCT) consolidation. Because of his high IgM serum level, three plasma exchange procedures were performed before treatment initiation. Induction treatment included bortezomib, dexamethasone, and thalidomide (bortezomib 1.3 mg/m^2^ on days 1, 4, 8, and 11 of each 28-day cycle; dexamethasone 40 mg/day once per week; thalidomide 100 mg/day) for a total of four 28-day courses. This induction regimen was followed by one cycle of cyclophosphamide (3 g/m^2^ for 1 day) for stem cell harvest, and then ASCT consolidation conditioned by melphalan 140 mg/m^2^ regimen (dose reduced according to age). Maintenance therapy with lenalidomide (10 mg/day) was planned in case of a good response.

The patient showed good tolerance to chemotherapy, with achievement of a good partial response. His IgM monoclonal spike was significantly reduced (1700 mg/L). He underwent ASCT in November 2019, reaching a very good partial remission, and maintenance treatment with lenalidomide is ongoing.

### Patient 2

Patient 2 was an 84-year-old Caucasian man with two brothers and one sister. He was married with one son and was a retired teacher. He was a smoker and consumed one drink of alcohol per day. He had a penicillin allergy and a clinical history of many comorbidities. He showed hypertension, chronic bronchopneumopathy, a known kidney injury, and a cardioembolic stroke episode related to chronic atrial fibrillation without clinical outcomes. Before his hematological diagnosis, he was receiving treatment with many chronic medications: valsartan/amlodipine for hypertension, salbutamole inhaled for chronic bronchopneumopathy, and bisoprolol and anticoagulation for atrial fibrillation. He experienced rapidly progressive fatigue, and basal blood laboratory test results showed a severe normocytic anemia (Hb 7.3 g/dl) with normal platelet and white blood cell counts. Upon admission to the emergency room, the patient’s blood pressure was 105/70 mmHg, and his pulse was 95 beats/minute with known atrial fibrillation rhythm. He had a mild fever with a body temperature of 38.2 °C. His clinical examination revealed that he had axillary bilateral enlarged adenopathy and palpable splenomegaly, 6-kg weight loss in the last few months, no night sweats, and no new-onset bone pain. His neurological assessment did not show any neurological impairment, just intense fatigue. Esophagogastroduodenoscopy and colonoscopy demonstrated jejunal angiodysplasia, so he was treated with iron support, which led to a partial recovery of his Hb level. Some months later, new worsening of anemia and progressive thrombocytopenia were observed (Hb 8.5 g/dl, platelets 40,000/mm^3^, with normal blood cell count of leukocytes 8350/mm^3^, neutrophils 3400/mm^3^). Extended blood laboratory test results showed an M-spike at protein electrophoresis (300 mg/L; 4.9%) with serum IgM level of 327 mg/L and elevated β_2_-microglobulin level of 5.6 mg/dl. His serum creatine level was stable in known chronic kidney disease (creatinine 1.54 mg/dl), and his liver function was normal.

To better define the clinical situation, a bone marrow biopsy was performed. Patient 2’s diagnosis was IgM MM with an increased number of clonal CD138^+^, IgM^+^, cyclin D1^+^, MUM1^+^, CD56^−/+^, CD79^−/+^, CD20^−^, CD19^−^, κ-light chain–negative plasma cells (30–40%) (Figs. 1e–h, 2b).

**Fig. 4:**
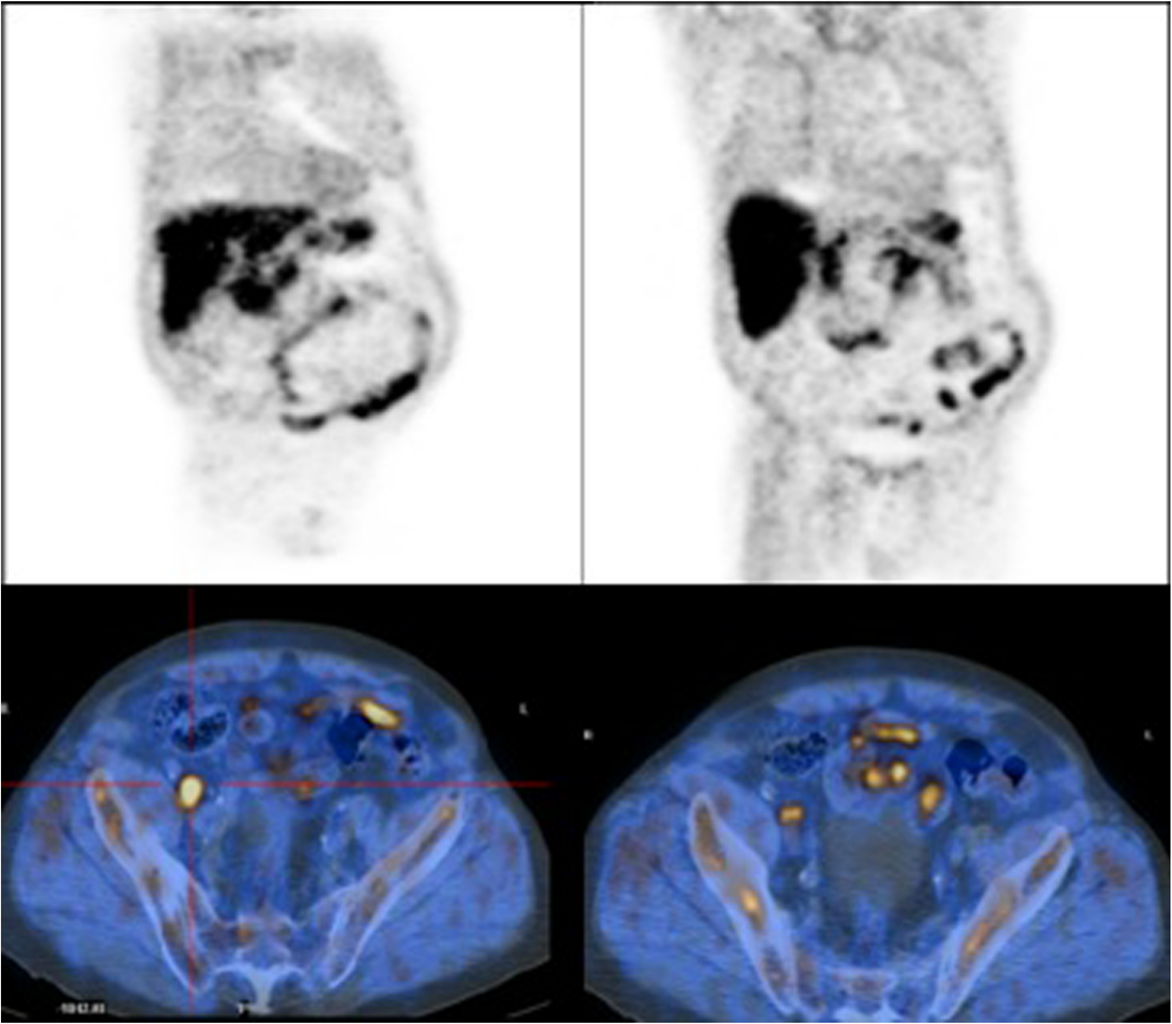
Positron emission tomography–computed tomography of patient 2

Positron emission tomography–computed tomography showed many enlarged perihepatic, peripancreatic, mesenteric lymph nodes (maximum diameter 6.6 cm). Splenomegaly was also observed (18 cm), while his liver test results were normal (Fig. 4).

Clinical features with enlarged lymph nodes and the absence of bone lesions were suggestive of WM, in contrast to the bone marrow biopsy results. To clarify the differential diagnosis, MYD88 L265P mutation was researched in peripheral blood and bone marrow aspirate samples, which both produced positive results (Fig. 3b). Considering the patient’s age and comorbidities, FISH analysis was not performed. For the same reasons, treatment with rituximab plus bendamustine seemed contraindicated, and the patient received rituximab, cyclophosphamide, and dexamethasone for six courses (rituximab 375 mg/m^2^ on day 1, cyclophosphamide 100 mg/m^2^ twice daily from day 1 to day 5, and dexamethasone 40 mg on day 1 of a 21-day cycle).

After the second therapy course, recovery of platelet level and Hb was observed, and the patient’s ultrasound scan showed a reduction of all abdominal lymph nodes and of his spleen diameter. The patient was in complete remission at 6-month follow-up after the end of treatment.

## Discussion and conclusions

A correct differential diagnosis between IgM MM and WM still represents a challenge for clinicians because of the small number of cases of IgM MM reported in the literature. Nevertheless, these two hematological entities require different treatments and have different outcomes, so a correct diagnosis is mandatory.

The two patients we describe in this report showed the same bone marrow diagnosis. However, clinical presentation and radiological analysis suggested different hematological diseases, demonstrating that biopsy alone is not sufficient for a correct final diagnosis.

Because of the rarity of IgM MM, few reports are published in the literature. The largest retrospective series, reported by Castillo *et al.* [7], comprised 134 cases of IgM MM that showed as pathognomonic features the presence of bone marrow clonal plasma cells CD38^+^ and/or CD138^+^, cyclin D1^+^, and t(11,14)-positive in almost 40% of cases. CD20 was positive in half of the patients. In these series, only 15 patients were tested for MYD88 L265P mutation, and all showed negative results. The clinical presentation of these patients usually met CRAB criteria, with no splenomegaly and/or adenopathy. Some patients presented with hyperviscosity symptoms. The same characteristics were shown in a smaller series of 21 cases of IgM MM at the Mayo Clinic [3].

In contrast, many reports underline the importance of MYD88 L256P mutation in patients with WM as the pathognomonic sign [5]. However, according to European Society for Medical Oncology clinical practice guidelines [8], MYD88 mutation alone cannot be considered diagnostic for WM. This mutation is also found in IgM MGUS and may also be found in other lymphomas, such as marginal zone lymphomas. Furthermore, about 5–10% of patients who fulfill diagnostic criteria for WM do not have the MYD88 L256P mutation [9].

Our patient 1 showed both a clinical presentation (no adenopathy and splenomegaly, no B symptoms, and no new-onset bone pain, but a moderate macrocytic anemia) and bone marrow histology suggestive of IgM MM. As expected, translocation t(11,14) was detected by FISH analysis, and the result of testing for the MYD88 L256P mutation was negative. On the basis of these multidimensional findings, a diagnosis of IgM MM was made. Our patient 2 showed a clinical presentation more suggestive of lymphoproliferative disease: fatigue and B symptoms with lymphadenopathies. Bone marrow histology suggested IgM MM. On the basis of these contrasting data between clinical and histological features, biological analysis was performed, and the result of testing for the MYD88 L256P mutation was positive. On the basis of these data, a diagnosis of WM was made.

Treatment changes according to diagnosis. With a final diagnosis of IgM MM, the treatment for young patients who are transplant eligible is composed of a bortezomib-based induction therapy (for example, bortezomib plus thalidomide plus dexamethasone), followed by ASCT and maintenance treatment [10]. Patients not eligible for high-dose chemotherapy can receive different regimens, such as bortezomib, melphalan, and prednisone. Our patient 1 was a candidate for ASCT. In the setting of a WM diagnosis, treatment is different and has been changing in recent years. It can be based on chemoimmunotherapy regimens, such as rituximab plus bendamustine in suitable patients or rituximab plus cyclophosphamide plus dexamethasone in unsuitable ones [11, 12]. Milder regimens can be used in frail patients. On the basis of the proven efficacy of Bruton tyrosine kinase inhibitors such as ibrutinib in MYD88 L256P–related hematological disease, ibrutinib is considered by the recent guidelines for treatment of WM in both first and further lines of therapy [8, 9]. Our patient 2 was considered unfit and underwent rituximab plus cyclophosphamide plus dexamethasone, with a good response.

In conclusion, IgM monoclonal gammopathy is a characteristic sign that can mask many different hematological diseases. A correct differential diagnosis between IgM MM and WM is a critical point in the management of patients with new-onset IgM monoclonal gammopathy. These patients should undergo a complete diagnostic workup comprising pathological, radiological, and serological examinations to establish the diagnosis and plan the most appropriate treatment in order to improve the prognosis.

## Data Availability

The data that support the findings in this report were collected from clinical/pathological/laboratories/radiologic reports and charts recorded in paper or informatics systems and are available from the corresponding author on reasonable request.

## Abbreviations

ASCT: Autologous stem cell transplant
CRAB: Hypercalcemia*,* renal failure*,* anemia*,* and bone lesions
FISH: Fluorescence *in situ* hybridization
Hb: Hemoglobin
IgM: Immunoglobulin M
MGUS: Monoclonal gammopathy of unknown significance
MM: Multiple myeloma
WM: Waldenström macroglobulinemia
